# Speed guidance strategy at intersections based on platoon recognition in connected and autonomous vehicles environments

**DOI:** 10.1371/journal.pone.0352282

**Published:** 2026-06-25

**Authors:** Shenzhen Ding, Zhengjun Wu, Fei Peng, Yanwei Xu, Xin Wang, Aihua Fan, Rongjun Zheng

**Affiliations:** 1 School of Civil Engineering and Communication, North China University of Water Resources and Electric Power, Zhengzhou, China; 2 Shandong Engineering Research Center of Intelligent Traffic Control and Guidance Technology for Public Security, Shandong Police College, Jinan, China; 3 School of Intelligent Transportation, Xuchang University, Xuchang, Henan, China; Beijing Institute of Technology, CHINA

## Abstract

To alleviate the issues of widespread traffic congestion and low passage efficiency at urban signalised intersections, which result in increased vehicle energy consumption, this paper proposes a speed guidance strategy based on platoon recognition in connected and autonomous vehicle (CAV) environments. The study focuses on vehicle platoons, considering the impact of varying CAV penetration rates, CAV aggregation intensity and the spatio-temporal distribution of mixed traffic flows. Six distinct platoon passage scenarios through intersections are defined, based on whether platoons encounter obstructions. Three distinct guidance strategies are proposed for these scenarios: acceleration guidance, deceleration guidance and platoon splitting. Finally, a case study on secondary development based on Vissim is conducted. The results show that the platoon-based speed guidance strategy reduces vehicle fuel consumption (from 3.152 to 0.600 L/s), delay time (from 9.22 to 3.79 s), and the number of stops (from 0.18 to 0.04 times) compared to no speed guidance Furthermore, the effectiveness of platoon-based speed guidance strategies varies with CAV penetration rates. As the CAV penetration rate approaches 0.7, the benefits to traffic of the guidance strategy become more apparent. The most significant reductions were observed in fuel consumption, delay time and the number of stops: 40%, 37% and 23%.

## 1. Introduction

It is becoming increasingly clear that congestion is a widespread problem at urban signal-controlled intersections. The operational efficiency of signalised intersections is therefore of critical importance to the performance and quality of the entire transport system. Disruptions to signal cycles result in vehicles exhibiting intermittent stop-and-go behaviour when traversing intersections. This has been shown to have a detrimental effect on travel efficiency by exacerbating traffic congestion. Furthermore, it has been demonstrated that this increase in congestion leads to higher energy consumption and exhaust emissions, produced by frequent acceleration and deceleration. Consequently, air pollution worsens. In order to effectively alleviate vehicle delays, reduce pollutant emissions and enhance the overall efficiency of the road network, it is imperative to analyse vehicle flow patterns at signalised intersections. Building upon this analysis through research into speed guidance strategies is of significant importance for improving intersection efficiency and advancing transportation sustainability.

Advances in CAV (Connected and Autonomous Vehicle, CAV) technologies have altered driving behaviours in comparison to traditional traffic environments, leading to systematic changes in traffic flow-related operational parameters. In a connected autonomous environment, CAVs can obtain real-time information about surrounding vehicles and road conditions, enabling them to adjust their driving state more precisely. This facilitates coordinated following, lane changes, and speed control, resulting in reduced headway distances, smoother acceleration and deceleration processes, and more collaborative travel strategies. These behavioural changes further alter parameters such as traffic flow, average speed, and critical density, while also impacting delay, the number of stops, energy consumption, and emission levels. As CAV penetration rates evolve, traffic flow stability and road capacity will undergo corresponding adjustments. Consequently, many researchers studying vehicle speed induction at signalised intersections now consider the impact of CAV integration. Furthermore, studies suggest that approximately 70% of vehicles on the road travel in platoons [1]. However, current research tends to focus on individual vehicles rather than on the tendency for them to form platoons at signalised intersections. This can result in models that fail to accurately reflect real-world traffic conditions and hinder the development of effective traffic management strategies.

This paper focuses on the environment of CAVs, particularly vehicle platoons. It identifies platoons approaching intersections by considering two metrics: headway time and speed difference. Based on the obstruction status of platoons at intersections, six platoon passage scenarios are categorised. After defining the speed guidance zone, tailored speed guidance strategies are designed for these scenarios. Finally, the designed speed guidance strategies are verified using a Python-based simulation of the Vissim software. The effectiveness of these strategies in different scenarios is then compared. The objective of the paper is to optimise the travel state of vehicles through cooperative platoon control, thereby enhancing the efficiency of intersection throughput, reducing fuel consumption, and lowering exhaust emissions.

In contradistinction to conventional studies on single-vehicle speed guidance, this paper focuses on connected autonomous driving environments. The integration of V2X data, alongside the consideration of two metrics (headway and speed difference), facilitates the identification of vehicle platoons approaching intersections. This shift in focus entails a transition from the consideration of individual vehicles to that of the platoon in its entirety, understood as a spatio-temporally continuous entity. This transition serves to enhance the coordination and execution efficiency of guidance commands, while concomitantly mitigating the occurrence of secondary disturbances. Such disturbances are typically caused by frequent stop-and-go cycles and inconsistent acceleration/deceleration in single-vehicle control. The present study employs an analytical approach to investigate the coupling relationships among the convoy’s arrival phase, length, and traffic signal states at intersections. This analysis enables the scientific categorization of six convoy passage scenarios. The system then establishes three control modes: namely, acceleration guidance, deceleration guidance and convoy segmentation. These form six refined speed guidance schemes, which are tailored to different scenarios. This creates a comprehensive, scenario-matched convoy coordination system that effectively addresses the limitations of single, fixed guidance strategies.

Finally, the designed speed guidance strategies are verified using a Python-based simulation of the Vissim software.A comprehensive comparison of traffic performance differences was conducted, encompassing multiple indicators such as travel efficiency (average delay and average number of stops), energy consumption, and exhaust emissions. This comparison was made under varying levels of CAV penetration, both with and without guidance strategies. The objective of the paper is to optimise the travel state of vehicles through cooperative platoon control, thereby enhancing the efficiency of intersection throughput, reducing fuel consumption, and lowering exhaust emissions. The paper establishes a theoretical basis for the precise management of urban signalised intersections, which is crucial for the development of intelligent urban transportation systems and the creation of sustainable, environmentally friendly transportation solutions.

## 2. Literature review

Traffic signal controls often result in interrupted driving patterns and queuing behaviour at junctions. This reduces traffic efficiency and increases vehicle emissions and fuel consumption. Therefore, it is important to implement reasonable speed guidance approaches before signalised intersections to smooth vehicle trajectories and mitigate urban traffic congestion, reducing energy consumption and minimising environmental pollution. This issue has attracted significant attention from researchers worldwide, resulting in extensive studies on vehicle speed guidance strategies for signalised intersections.

Although research has focused on coordinated control and trajectory optimisation for CAVs, existing models often rely on unrealistic assumptions, such as high CAV penetration rates, stable and delay-free communication, and complete compliance among all road users. These assumptions differ significantly from real-world operational conditions. Furthermore, the associated methodologies are often computationally complex and inefficient, rendering them unsuitable for real-time intersection control applications. Additionally, many studies overlook critical behavioural factors in mixed traffic environments. These include HDV (Human-driven Vehicle, HDV) response mechanisms to CAV-generated instructions and human–machine interaction issues, such as user acceptance and behavioural compliance. Such omissions compromise the effectiveness and reliability of these strategies when applied in practice. For example, Jiang et al. [2] proposed a method of segmented cooperative control for managing the flow of urban road traffic in a connected vehicle environment. This approach uses a two-tier optimisation model to coordinate the optimisation of both arterial and intersection traffic flow. The simulation results show that the proposed ML-SLSG strategy successfully reduces lane-changing distances. Meanwhile, the longitudinal trajectory optimisation model decreases the average intersection delay by between 5.9% and 8.0%. Niu [3] developed a crossing decision model for mixed traffic flows. This model uses a cellular automata framework and considers the characteristics of HDVs, as well as the real-time reception of information by CAVs. Wang et al. [4] introduced a coordinated control method that integrates traffic signal timing and vehicle trajectory optimisation. This method is based on platooning and trajectory planning, and is intended for use in a CAV mixed-traffic environment. They used a genetic algorithm to optimise the timing of the upper-level signal and the trajectories of the vehicles. Liu et al. [5] investigated the optimisation of trajectories for individual autonomous vehicles in mixed CAV and HDV environments. Their simulation results suggest that optimising the trajectories of autonomous vehicles can influence the behaviour of vehicles driven by humans, thereby reducing the average travel time. Liu et al. [6] proposed a cooperative adaptive cruise control strategy for CAVs to increase the likelihood of crossing intersections without stopping. This mitigates the fuel consumption associated with acceleration and deceleration in stop-and-go scenarios. Meanwhile, Baby et al. [7] introduced a novel, robust advisory control framework based on model predictive control (MPC) to improve the fuel efficiency of connected vehicles in various urban traffic scenarios. In this framework, CAVs provide recommended commands for HDVs to follow. This improves the speed profile of both vehicle types, thereby enhancing the overall fuel economy. Yao et al. [8] used various car-following models to capture the behavioural characteristics of different driving modes in mixed traffic. Their results demonstrate that CAVs can significantly reduce fuel consumption. Tajalli et al. [9] developed a distributed optimisation and coordination algorithm to dynamically optimise the speed of CAVs in urban road networks. Simulation validation confirmed that the model effectively reduces travel time, average delay, and the number of stops at intersections.

Moreover, although some studies have examined traffic signal control and capacity optimisation, these studies have significant limitations. It is clear that many models are designed for specific intersection geometries or static traffic conditions. This results in models exhibiting limited generalisability and poor adaptability to diverse intersection layouts. Furthermore, conflicts arise between optimisation objectives, such as the exclusive pursuit of capacity maximisation or delay minimisation, which can negatively impact other performance metrics, including fuel economy, emission control and traffic equity. There is clearly a lack of effective mechanisms for systematic multi-objective optimisation. For example, Liang et al. [10] proposed a Multi-Traffic Flow Aggregation Control Model (MTF-ACM) for CAV mixed-traffic flow based on multi-agent systems (MAS). The model under discussion has been developed to support platooning strategies that depend on vehicle-to-vehicle technology. The simulation results suggest that the MTF-ACM achieves optimal performance with a CAV penetration rate of 60%. In their seminal paper, Wu et al. [11] proposed a signal control strategy incorporating dedicated phases for autonomous vehicles. This strategy is further enhanced by a capacity prediction model based on queuing theory for these phases. A mixed-integer nonlinear programming model was formulated to maximise capacity while taking into account constraints such as signal timing, lane configuration and traffic flow allocation. Yue [12] proposed a signal optimisation method for intersections with left-turn and through waiting areas. This method optimises both signal timing and vehicle trajectories, thereby improving operational smoothness at such intersections. Feng et al. [13] developed a reinforcement learning–based model to increase intersection throughput. This model incorporates leading vehicle acceleration and safe longitudinal following distances. The study’s findings suggest that the proposed approach effectively reduces energy consumption and the number of stops when acceleration and deceleration rates are incorporated as optimisation objectives. Lin et al. [14] established models for average intersection delay, average queue length, total delay, and vehicle emissions. Moreover, a novel optimisation model for arterial coordination control was derived. The researchers also proposed a traffic signal optimisation algorithm based on fuzzy control theory and a differential evolution algorithm (FASM-MDEA).

Furthermore, studies focusing on eco-driving, energy conservation and emission reduction face inherent challenges. The effectiveness of these strategies depends on the availability of precise, real-time information. However, little research has been conducted into how they perform in the event of sensing errors, communication delays and information uncertainty. There is a fundamental conflict between optimising microscopic vehicle trajectories and maintaining the stability of macroscopic traffic flow. Optimising speed profiles for individual vehicle energy efficiency can lead to reduced travel speed and the formation of moving bottlenecks in high-flow scenarios, causing rear-end congestion. This congestion can offset or even exacerbate energy consumption at the system level. Consequently, further validation is required to ascertain the applicability and effectiveness of such strategies in complex urban road networks. For example, Huang et al. [15] developed an eco-driving strategy based on queue length at real-world intersections. The researchers developed three eco-driving modes — “accelerate-coast”, “decelerate-coast” and “constant speed-coast” — and created mathematical models to correspond with each one. The proposed approach is characterised by its operational simplicity and its ability to encourage eco-driving, which leads to reduced energy consumption and emissions. Dong et al. [16] estimated real-time queue lengths using data collected in a connected vehicle environment, incorporating them into an energy consumption optimisation model. The simulation results indicated a 30% improvement in energy utilisation efficiency under the eco-driving strategy. Sun [17] formulated eco-driving as a data-driven, chance-constrained optimisation problem that accounted for uncertainties in traffic signal timing, speed limits and minimum headway. They solved this problem using dynamic programming. Their findings showed a 40% decrease in vehicle energy dissipation. Chen et al. [18] designed a GLOSA system for public transport (B-GLOSA) that integrated an instantaneous fuel consumption model, a vehicle dynamics model and the relationship between traffic signals, vehicle speed and distance to the intersection. They demonstrated that B-GLOSA reduced fuel consumption and travel time by 22.1% and 6.1%, respectively, compared to driving without speed guidance, by formulating a vehicle trajectory optimisation problem. Zhao et al. [19] proposed a dynamic eco-driving strategy for vehicles crossing two consecutive intersections. This strategy involved implementing different speed guidance policies before and after the first intersection, independently calculating energy consumption and optimising vehicle trajectories. The study’s findings demonstrated that the multi-intersection strategy was more energy efficient than the single-intersection optimisation.

Research on the optimization of vehicle platoon recognition and speed guidance strategies at intersections in a networked autonomous driving environment relies on platoon coordination control, vehicle-to-vehicle communication, intelligent decision-making algorithms, and vehicle-to-infrastructure (V2I) technologies. Existing research in this field has already provided theoretical and technical references from multiple perspectives. For example, Nandhini et al. [20] proposed a methodology for the implementation of clustering strategies in a platoon, with the objective of achieving string stability by minimising disturbances and variations in vehicle speed and position. The study involves an in-depth analysis of clustering algorithms to identify the most suitable approach for integration into vehicle platooning, specifically for network analysis purposes. Zhang et al. [21] proposed an information transmission method based on LED matrix and monocular vision. This is achieved through the use of an LED matrix embedded in the leading vehicle′s taillight for communication. Experiments reveal that the approach can facilitate vehicle-to-vehicle information transmission even when network conditions are completely absent, thereby enhancing driving safety. Irshayyid et al. [22] provided a comprehensive review of recent advancements in the application of DRL to highway lane change, ramp merge, and platoon coordination. such as the state representation methods that capture interactive dynamics critical for safe and efficient merging and the reward formulations that manage key metrics. this review can guide future research toward realizing the potential of DRL for automated driving in complex traffic under uncertainty. Pan et al. [23] proposed a V2X-based lane change decision method for intelligent connected electric vehicles. They constructed an energy consumption cloud model and adopted the PSO-LSTM model to predict the speed of preceding vehicles. They also established an AHP-based lane change decision method. The simulation results showed that this method can select the most economical lane and reduce energy consumption by up to 27.2% in scenarios involving continuous lane changes.

## 3. Methodology

Currently, most research on intersection speed guidance focuses on individual vehicles. While single-vehicle speed guidance performs well under low traffic volumes, its effectiveness diminishes under high-flow conditions, particularly in urban areas. In such contexts, vehicle interactions intensify, car-following behaviours become more commonplace, and the driving behaviour of a preceding vehicle has a significant influence on those behind. It is important to note that ignoring these interactions and applying speed guidance exclusively to individual vehicles may result in the guided vehicle failing to pass through the junction while the lights are green. Consequently, the intended outcomes may not be achieved. The present study therefore proposes a platoon-oriented speed guidance strategy for signalised intersections, with the aim of addressing this issue. A “platoon” is defined as a group of multiple vehicles.

The intersection speed guidance strategy proposed in this paper is based on platoon recognition. As a core technology in intelligent transportation and automated driving systems, the identification of platoons has been the subject of extensive study in recent years, with research focusing on model architecture, multi-modal information fusion and scenario adaptability. Scholars have adopted various technical approaches and application emphases. In line with the specific context and objectives of this study and considering the spatiotemporal characteristics of mixed traffic flows, it is hypothesised that, in a connected and autonomous driving environment, all CAVs will be capable of sharing real-time information, including vehicle position, speed and signal timing, with each other and with roadside units with negligible communication delay.

This study uses previous research [24] to identify platoons. The process comprises three primary steps: platoon discrimination; estimation of platoon length; and estimation of the timings of the head and tail vehicles. The robustness of the vehicle platoon identification method and its core parameters (platoon discrimination; estimation of platoon length; and estimation of the timings of the head and tail vehicles) used in this paper has been thoroughly demonstrated in previous research. The previous research utilised a comprehensive experimental approach encompassing multiple sets of parameter sensitivity tests and control experiments. These experiments were designed to emulate a range of mixed-traffic flow conditions, account for vehicle heterogeneity, and incorporate driving disturbance scenarios. These experiments validated the stability and reliability of the selected lead time and adjacent vehicle speed difference parameters across various scenarios. This provides a solid foundation for the subsequent research and implementation of intersection speed guidance strategies. In this paper, the need to repeat the relevant sensitivity analyses is eliminated. This method uses time headway and speed differences between consecutive vehicles as identification metrics to incorporate the spatiotemporal features of both CAV and HDV in mixed traffic. This enables the construction of a platoon recognition model, providing a foundation for the subsequent speed guidance strategy.

### 3.1. Platoon performance at intersections

In a connected and automated driving environment, vehicles approaching an intersection have the capacity to exchange information via onboard units (OBUs) and roadside units (RSUs). Vehicles receive real-time signal phase and timing (SPaT) information from the junction, while the infrastructure acquires dynamic vehicle data, such as position and speed. In accordance with established methodologies for identifying platoons in mixed traffic flow contexts, vehicles approaching an intersection can be categorised and identified as platoons. Following the definition of a speed guidance zone, the system determines whether the lead and tail vehicles of a platoon can navigate the intersection successfully without decelerating, using the vehicles’ current position and speed in conjunction with SPaT data. Depending on the platoon’s impedance status at the intersection, this paper categorises crossing scenarios into six types.

#### Scenario 1: Unimpeded Platoon.

In this scenario, the traffic signal remains green for the entire duration of the platoon’s passage. The platoon leader and the tail vehicle can both pass through the intersection at their current speed without decelerating or stopping.

#### Scenario 2: Partially Impeded Platoon — Tail Vehicle Can Pass via Acceleration.

In this scenario, the platoon is partially impeded; the platoon leader arrives at the intersection under a green signal at the current speed, but the tail vehicle will encounter a red signal. However, the tail vehicle can avoid stopping by accelerating to the speed limit and passing through the junction.

#### Scenario 3: Partially Impeded Platoon — Tail Vehicle Cannot Pass via Acceleration.

In this scenario, the platoon is partially impeded. Although the platoon leader passes under a green signal, the platoon tail vehicle arrives during the red phase. Even after accelerating to the maximum speed limit, the tail vehicle cannot clear the intersection without stopping.

#### Scenario 4: Fully Impeded Platoon — Entire Platoon Can Pass via Acceleration.

In this scenario, the entire platoon is fully impeded, arriving during the red phase. However, both the platoon leader and the platoon tail vehicle can avoid stopping by accelerating to the maximum allowed speed and passing through the intersection.

#### Scenario 5: Fully Impeded Platoon — Platoon Leader Can Pass via Acceleration, Platoon Tail Cannot.

In this scenario, the entire platoon is fully impeded by the red signal. While the platoon leader can pass without stopping by accelerating to the speed limit, the platoon tail vehicle cannot clear the intersection in time, even under maximum acceleration.

#### Scenario 6: Fully Impeded Platoon — Entire Platoon Cannot Pass via Acceleration.

In this scenario, the platoon is fully impeded and arrives entirely during the red phase. Neither the platoon leader nor the platoon tail vehicle can pass through the intersection without stopping, even when accelerating to the maximum speed limit.

**Fig 1:**
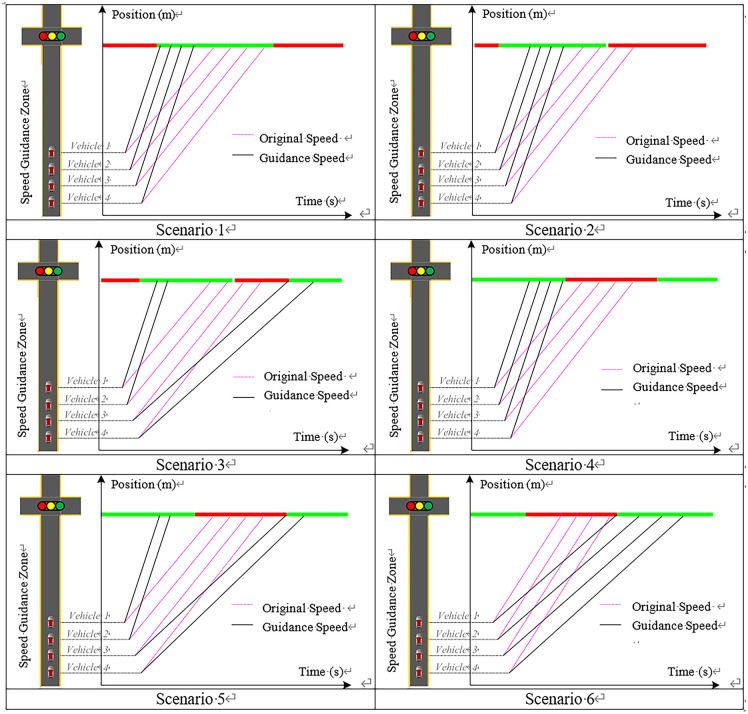
Time-space Diagrams of the Six Situations.

A schematic time-space diagram illustrating the six platoon scenarios at the signalized intersection is presented in Fig 1.

The transitions between the six traffic scenarios and their real-time detection is contingent on the relationship between the arrival times of the lead and trailing vehicles at the intersection, while maintaining their current speeds, and the start and end times of the traffic lights. This is combined with a dynamic assessment of their ability to accelerate to maximum speed. The following outline delineates the process: The distinction between Scenario 1 and Scenarios 2 and 3 lies in the configuration of the rear vehicle’s encounter with a red light at the time of arrival; Scenarios 2 and 3 are further differentiated based on the ability of the vehicle to clear the intersection before the green light ends after accelerating to maximum speed; Scenarios 4–6 address scenarios where the lead vehicle encounters a red light immediately, and are classified sequentially based on the ability of the lead and rear vehicles to clear the intersection after accelerating. These transitions can be determined through real-time threshold analysis using vehicle position, speed, and traffic signal phase differences.

### 3.2. Platoon speed guidance strategies for different scenarios

Prior to implementing speed guidance, the following basic assumptions are defined based on the six platoon scenarios proposed above:

(1) When a platoon enters the speed guidance zone, the vehicles generally do not overtake or change the platoon’s lane configuration.
(2) Onboard units (OBUs) of CAVs, roadside units (RSUs) and the control centre can communicate with each other in real time. CAVs can share real-time information, such as vehicle position, speed and signal status, with each other and with traffic signals.
(3) Interaction delays between vehicles and between vehicles and roadside equipment are negligible.
(4) The road surface is assumed to be flat and the effect of the road gradient is ignored.
(5) Vehicle arrivals follow a Poisson distribution.

As this paper focuses on urban arterial roads and aims to provide speed guidance for platoons travelling on them, it is first necessary to define the length of the speed guidance zone at the entrance to the road. The guidance zone length should be determined according to the following two principles:

(1) The minimum length of the guidance zone must ensure that, regardless of the platoon’s entry speed, there is sufficient time for vehicles to adjust their driving status.
(2) The maximum guidance zone length should ensure that vehicles within the platoon can traverse the intersection within one signal cycle while adhering to the speed limit of the road segment.

Based on these principles, the length range of the speed guidance zone in this paper is estimated using Equation(1).


$$\begin{array}{c} L\subset \left[max\left\{\frac{\overset{\text{2}}{\underset{max}{V}}-\overset{\text{2}}{\underset{min}{V}}}{2a},\frac{\overset{\text{2}}{\underset{max}{V}}-\overset{\text{2}}{\underset{min}{V}}}{2d}\right\},CV_{min}\right] \end{array}$$ (1)

In the equation, *L* is the length of the speed guidance zone, *V*_*min*_ and *V*_*max*_ are the minimum and maximum speed limits of the road segment, *a* is the absolute value of comfortable acceleration perceived by the driver, *d* is the absolute value of comfortable deceleration perceived by the driver, and *C* is the signal cycle length.

In traffic flow theory, traffic volume is expressed as the product of traffic density and vehicle speed. To enhance intersection capacity, it is essential to implement traffic control methods that maximise vehicle density and speed at the intersection. To this end, roadside units (RSUs) are installed at intersections to collect travel information from vehicles within the speed guidance zone and transmit it to a central controller. CAVs are equipped with OBUs that acquire real-time operational status and upload it to the central controller, while also receiving control commands from it. When a vehicle enters the guidance zone, the central controller determines its speed and position in real time, and the vehicle can read the current signal status. If both the lead and tail vehicles in a platoon are capable of negotiating the intersection after accelerating to the stipulated speed limit, the system will instruct the platoon to accelerate to the limit speed and then maintain a constant velocity. This will enhance the efficiency with which the intersection is traversed. If a platoon of vehicles is unable to successfully pass an intersection after accelerating to the stipulated speed limit, the system determines whether they can successfully pass during the subsequent green interval at their current speed. If so, the system provides guidance to the vehicles to maintain a constant speed. Conversely, if the vehicles cannot pass during the green interval, the system instructs them to decelerate and stop to wait.

The platoon speed guidance strategy outlined in this paper comprises three modules. The first module is the acceleration guidance strategy, which enhances overall platoon traffic efficiency by dynamically regulating the acceleration of the leading vehicle while ensuring safe inter-vehicle spacing. Secondly, the deceleration guidance strategy anticipates potential collision risks in conflict zones and implements speed reduction. Thirdly, the platoon splitting strategy divides oversized platoons into several sub-platoons according to signal phase characteristics. This achieves precise spatiotemporal resource allocation through phase offset optimisation. These modules operate under a collaborative control framework, dynamically adjusting vehicle states to ensure efficient and safe passage through intersections for platoons.

#### 3.2.1. Acceleration guidance strategy.

The control objective is to minimize the time for vehicles in the platoon to accelerate to the intersection stop line. Its mathematical expression is shown in Equation(2).


$$\begin{array}{c} minT_{i_{\text{1}}}=T_{i_{\text{0}}}+\frac{v_{i_{\text{1}}}-v_{i_{\text{0}}}}{a}+\frac{L-\frac{\overset{\text{2}}{\underset{i_{\text{1}}}{v}}-\overset{\text{2}}{\underset{i_{\text{0}}}{v}}}{2a}}{v_{i_{\text{1}}}} \end{array}$$ (2)

The constraints are as follows:


$$\begin{array}{c} s.t.\left\{ \begin{array}{c} @c@T_{(i-1{)}_{\text{1}}}+T_{s}\leq T_{I_{\text{1}}}\leq T_{g}\\ S\geq \frac{\left|\overset{\text{2}}{\underset{i_{\text{1}}}{v}}-\overset{\text{2}}{\underset{(i-1{)}_{\text{1}}}{v}}\right|}{2a}+\tau v_{i_{\text{1}}}+l\\ v_{i_{\text{1}}}\leq V_{max} \end{array}\right. \end{array}$$

In the equation, $T_{i_{\text{0}}}$ is the time when vehicle i enters the control zone, $T_{i_{\text{1}}}$ is the time when vehicle *i* arrives at the intersection stop line, $V_{i_{\text{0}}}$ is the speed when vehicle *i* enters the control zone, $V_{i_{\text{1}}}$ is the speed of vehicle *i* when vehicle *i* arrives at the intersection stop line, S is the safe headway distance between consecutive vehicles*, T*_*s*_ is the minimum time headway*, T*_*g*_ is the green end time, l is the vehicle length*,τ* is the total loss time, $v_{(i-1{)}_{\text{1}}}$ is the speed when vehicle *i-1* arrives at the intersection stop line, and $T_{(i-1{)}_{\text{1}}}$ is the time when vehicle *i-1* arrives at the intersection stop line.

#### 3.2.2. Deceleration guidance strategy.

The deceleration guidance strategy is activated when a vehicle in the platoon reaches the stop line at its current velocity and encounters a red signal. This is defined as the point at which the vehicle cannot pass the intersection within the current green phase, even after accelerating to the maximum permitted speed. The strategy then directs the vehicle to pass during the subsequent green phase. The objective of deceleration control is to maximise the speed of each vehicle in the platoon as it passes the stop line. After this module has been executed, vehicles in the platoon can choose to decelerate in order to pass the intersection at a reduced speed or to decelerate to a complete stop.

Assuming that the travel time of a vehicle in the platoon through the speed guidance zone is $t_{i}$, it can be estimated using Equation(3).


$$\begin{array}{c} t_{i}=\frac{v_{i_{\text{0}}}-v_{i_{\text{1}}}}{d}+\frac{L-\frac{\overset{\text{2}}{\underset{i_{\text{0}}}{v}}-\overset{\text{2}}{\underset{i_{\text{1}}}{v}}}{2d}}{v_{i_{\text{1}}}}=T_{i_{\text{1}}}-T_{i_{\text{0}}} \end{array}$$ (3)

After transformation, the objective function of the deceleration control module is given in Equation(4).


$$\begin{array}{c} v_{i_{\text{1}}}=v_{i_{\text{0}}}-dt_{i}+\sqrt{d^{\text{2}}\overset{\text{2}}{\underset{i}{t}}-2dt_{i}v_{i_{\text{0}}}+2dL} \end{array}$$ (4)

The constraints are as follows:


$$\begin{array}{c} s.t.\left\{ \begin{array}{c} @l@T_{g}\leq T_{i_{\text{0}}}+\frac{V_{max}-v_{i_{\text{0}}}}{a}+\frac{L-\frac{\overset{\text{2}}{\underset{max}{V}}-\overset{\text{2}}{\underset{i_{\text{0}}}{v}}}{2a}}{V_{max}}\\ T_{i_{\text{1}}}\geq T_{r}\\ T_{i_{\text{1}}}\geq T_{(i-1{)}_{\text{1}}}\\ S\geq \frac{\left|\overset{\text{2}}{\underset{i_{\text{1}}}{v}}-\overset{\text{2}}{\underset{(i-1{)}_{\text{1}}}{v}}\right|}{2d}+\tau v_{i_{\text{1}}}+l\\ V_{min}\leq v_{i_{\text{1}}}\leq V_{max} \end{array}\right. \end{array}$$

In the equation, *T*_*r*_ is the end time of the red phase at the intersection signal, with the other parameters consistent with prior definitions.

#### 3.2.3. Platoon splitting strategy.

This module is initiated when the platoon encounters a fully or partially obstructed intersection. When activated, some of the vehicles in the platoon accelerate to the maximum speed limit to pass through the intersection, while the rest are transferred to the deceleration control module. During the platoon splitting process, the control centre calculates the vehicle index corresponding to the split point, using real-time driving information. Acceleration control is then applied to the preceding vehicles and deceleration control to the following vehicles.

As outlined in the preceding sections, Table 1 provides an overview of the proposed platoon speed guidance strategies for the six platoon-crossing scenarios. The following strategies are based on the three modules described above. As shown in the provided table, the subscripts h and r refer to the platoon leader and tail vehicle, respectively. Meanwhile, the subscripts 0 and 1 indicate when the platoon reaches the speed guidance zone and the intersection stop line, respectively. The subscript n denotes the index of the vehicle at the split point during platoon splitting. After the manoeuvre has been executed, vehicle n takes on the role of leader for the residual platoon.

**Table 1:** Platoon speed guidance strategies for the six situations.

Platoon Crossing Scenario	Executed Module	Mathematical Expression	
		Objective Function	Constraints
Scenario 1	Acceleration Control	$minT_{h_{\text{1}}}=T_{h_{\text{0}}}+\frac{v_{h_{\text{1}}}-v_{h_{\text{0}}}}{a}+\frac{L-\frac{\overset{\text{2}}{\underset{h_{\text{1}}}{v}}-\overset{\text{2}}{\underset{h_{\text{0}}}{v}}}{2a}}{v_{h_{\text{1}}}}$	$\begin{array}{c} T_{(h-1{)}_{\text{1}}}+T_{s}\leq T_{h_{\text{1}}}\leq T_{g}\\ S\geq \frac{\left|\overset{\text{2}}{\underset{h_{\text{1}}}{v}}-\overset{\text{2}}{\underset{(h-1{)}_{\text{1}}}{v}}\right|}{2a}+\tau v_{h_{\text{1}}}+l\\ v_{h_{\text{1}}}\leq V_{max} \end{array}$
Scenario 2	Acceleration Control	$minT_{r_{\text{1}}}=T_{r_{\text{0}}}+\frac{v_{r_{\text{1}}}-v_{r_{\text{0}}}}{a}+\frac{L-\frac{\overset{\text{2}}{\underset{r_{\text{1}}}{v}}-\overset{\text{2}}{\underset{r_{\text{0}}}{v}}}{2a}}{v_{r_{\text{1}}}}$	$\begin{array}{c} T_{(r-1{)}_{\text{1}}}+T_{s}\leq T_{r_{\text{1}}}\leq T_{g}\\ T_{h_{\text{1}}}\geq T_{(h-1{)}_{\text{1}}}+T_{s}\\ S\geq \frac{\left|\overset{\text{2}}{\underset{r_{\text{1}}}{v}}-\overset{\text{2}}{\underset{(r-1{)}_{\text{1}}}{v}}\right|}{2a}+\tau v_{r_{\text{1}}}+l\\ S\geq \frac{\left|\overset{\text{2}}{\underset{h_{\text{1}}}{v}}-\overset{\text{2}}{\underset{(h-1{)}_{\text{1}}}{v}}\right|}{2a}+\tau v_{h_{\text{1}}}+l\\ v_{r_{\text{1}}}\leq V_{max} \end{array}$
Scenario 3	Platoon Splitting	$minT_{h_{\text{1}}}=T_{h_{\text{0}}}+\frac{v_{h_{\text{1}}}-v_{h_{\text{0}}}}{a}+\frac{L-\frac{\overset{\text{2}}{\underset{h_{\text{1}}}{v}}-\overset{\text{2}}{\underset{h_{\text{0}}}{v}}}{2a}}{v_{h_{\text{1}}}}$	First m Vehicles in the Platoon: $\begin{array}{c} T_{(h-1{)}_{\text{1}}}+T_{s}\leq T_{h_{\text{1}}}\leq T_{g}\\ S\geq \frac{\left|\overset{\text{2}}{\underset{h_{\text{1}}}{v}}-\overset{\text{2}}{\underset{(h-1{)}_{\text{1}}}{v}}\right|}{2a}+\tau v_{h_{\text{1}}}+l\\ v_{h_{\text{1}}}\leq V_{max} \end{array}$
		Remaining Vehicles in the Platoon: $maxv_{n_{\text{1}}}=v_{n_{\text{0}}}-dt_{n}+\sqrt{d^{\text{2}}\overset{\text{2}}{\underset{n}{t}}-2dt_{n}v_{n_{\text{0}}}+2dL}$	Remaining Vehicles in the Platoon: $\begin{array}{c} T_{g}\leq T_{n_{\text{0}}}+\frac{V_{max}-v_{n_{\text{0}}}}{a}+\frac{L-\frac{\overset{\text{2}}{\underset{max}{V}}-\overset{\text{2}}{\underset{n_{\text{0}}}{v}}}{2a}}{V_{max}}\\ T_{n_{\text{1}}}\geq T_{r}\\ T_{n_{\text{1}}}\geq T_{(n-1{)}_{\text{1}}}\\ S\geq \frac{\left|\overset{\text{2}}{\underset{n_{\text{1}}}{v}}-\overset{\text{2}}{\underset{(n-1{)}_{\text{1}}}{v}}\right|}{2d}+\tau v_{n_{\text{1}}}+l\\ V_{min}\leq v_{n_{\text{1}}}\leq V_{max} \end{array}$
Scenario 4	Acceleration Control	$minT_{r_{\text{1}}}=T_{r_{\text{0}}}+\frac{v_{r_{\text{1}}}-v_{r_{\text{0}}}}{a}+\frac{L-\frac{\overset{\text{2}}{\underset{r_{\text{1}}}{v}}-\overset{\text{2}}{\underset{r_{\text{0}}}{v}}}{2a}}{v_{r_{\text{1}}}}$	$\begin{array}{c} T_{(r-1{)}_{\text{1}}}+T_{s}\leq T_{r_{\text{1}}}\leq T_{g}\\ T_{h_{\text{1}}}\geq T_{(h-1{)}_{\text{1}}}+T_{s}\\ S\geq \frac{\left|\overset{\text{2}}{\underset{r_{\text{1}}}{v}}-\overset{\text{2}}{\underset{(r-1{)}_{\text{1}}}{v}}\right|}{2a}+\tau v_{r_{\text{1}}}+l\\ S\geq \frac{\left|\overset{\text{2}}{\underset{h_{\text{1}}}{v}}-\overset{\text{2}}{\underset{(h-1{)}_{\text{1}}}{v}}\right|}{2a}+\tau v_{h_{\text{1}}}+l\\ v_{r_{\text{1}}}\leq V_{max} \end{array}$
Scenario 5	Platoon Splitting	$minT_{h_{\text{1}}}=T_{h_{\text{0}}}+\frac{v_{h_{\text{1}}}-v_{h_{\text{0}}}}{a}+\frac{L-\frac{\overset{\text{2}}{\underset{h_{\text{1}}}{v}}-\overset{\text{2}}{\underset{h_{\text{0}}}{v}}}{2a}}{v_{h_{\text{1}}}}$	First m Vehicles in the Platoon: $\begin{array}{c} T_{(h-1{)}_{\text{1}}}+T_{s}\leq T_{h_{\text{1}}}\leq T_{g}\\ S\geq \frac{\left|\overset{\text{2}}{\underset{h_{\text{1}}}{v}}-\overset{\text{2}}{\underset{(h-1{)}_{\text{1}}}{v}}\right|}{2a}+\tau v_{h_{\text{1}}}+l\\ v_{h_{\text{1}}}\leq V_{max} \end{array}$
		Remaining Vehicles in the Platoon: $maxv_{n_{\text{1}}}=v_{n_{\text{0}}}-dt_{n}+\sqrt{d^{\text{2}}\overset{\text{2}}{\underset{n}{t}}-2dt_{n}v_{n_{\text{0}}}+2dL}$	Remaining Vehicles in the Platoon: $\begin{array}{c} T_{(r-1{)}_{\text{1}}}+T_{s}\leq T_{r_{\text{1}}}\leq T_{g}\\ T_{h_{\text{1}}}\geq T_{(h-1{)}_{\text{1}}}+T_{s}\\ S\geq \frac{\left|\overset{\text{2}}{\underset{r_{\text{1}}}{v}}-\overset{\text{2}}{\underset{(r-1{)}_{\text{1}}}{v}}\right|}{2a}+\tau v_{r_{\text{1}}}+l\\ S\geq \frac{\left|\overset{\text{2}}{\underset{h_{\text{1}}}{v}}-\overset{\text{2}}{\underset{(h-1{)}_{\text{1}}}{v}}\right|}{2a}+\tau v_{h_{\text{1}}}+l\\ v_{r_{\text{1}}}\leq V_{max} \end{array}$
Scenario 6	Deceleration Control	$maxv_{h_{\text{1}}}=v_{h_{\text{0}}}-dt_{h}+\sqrt{d^{\text{2}}\overset{\text{2}}{\underset{h}{t}}-2dt_{h}v_{h_{\text{0}}}+2dL}$	$\begin{array}{c} T_{g}\leq T_{h_{\text{0}}}+\frac{V_{max}-v_{h_{\text{0}}}}{a}+\frac{L-\frac{\overset{\text{2}}{\underset{max}{V}}-\overset{\text{2}}{\underset{h_{\text{0}}}{v}}}{2a}}{V_{max}}\\ T_{h_{\text{1}}}\geq T_{r}\\ T_{h_{\text{1}}}\geq T_{(h-1{)}_{\text{1}}}\\ S\geq \frac{\left|\overset{\text{2}}{\underset{h_{\text{1}}}{v}}-\overset{\text{2}}{\underset{(h-1{)}_{\text{1}}}{v}}\right|}{2d}+\tau v_{h_{\text{1}}}+l\\ V_{min}\leq v_{h_{\text{1}}}\leq V_{max} \end{array}$

To facilitate a clear understanding, the formulas listed in the table above are briefly explained below:

Scenario 1: The objective function minimizes the time it takes for the rear vehicle to reach the stop line. The constraints ensure that the rear vehicle crosses the stop line before the green light ends, while maintaining a safe distance S between vehicles and ensuring that speeds do not exceed the speed limit Vmax.

Scenario 2: The objective function is used to minimize the time it takes for the rear vehicle to reach the stop line. Constraint $T_{g}\leq T_{n_{\text{0}}}+\frac{V_{\text{max}}-V_{n_{\text{0}}}}{a}+\frac{L-\frac{\overset{\text{2}}{\underset{\text{max}}{V}}-\overset{\text{2}}{\underset{n_{\text{0}}}{V}}}{\text{2}a}}{V_{\text{max}}}$ ensures that the rear vehicle crosses the stop line before the green light ends while maintaining a safe following distance from the vehicle ahead; constraint $T_{h}\geq T_{(h-\text{1}{)}_{\text{1}}}+T_{s}$ ensures that the lead vehicle maintains a proper following order relative to the vehicle ahead; and the two safety distance inequalities S, along with the speed limit $v_{n}\leq V_{\text{max}}$, ensure that the rear vehicle does not rear-end another vehicle or exceed the speed limit during acceleration.

Scenario 3: The formula system is divided into two parts: For the front portion of the queue that can pass through the green light after the split, the objective function minimizes the arrival time of the lead vehicle (the vehicle at the split point) at the stop line; for the remaining portion of the queue that cannot pass through the current green light, constraint $T_{g}\leq T_{n_{\text{0}}}+\frac{V_{\text{max}}-V_{n_{\text{0}}}}{a}+\frac{L-\frac{\overset{\text{2}}{\underset{\text{max}}{V}}-\overset{\text{2}}{\underset{n_{\text{0}}}{V}}}{\text{2}a}}{V_{\text{max}}}$ indicates that even traveling at maximum acceleration, the vehicles cannot arrive before the green light ends, while constraint $T_{n}\geq T_{r}$ ensures that vehicles may not proceed until after the red light ends. $T_{n}\geq T_{(n-\text{1}{)}_{\text{1}}}$ maintains the following order. The safety distance inequality uses deceleration d in conjunction with speed limit $V_{\text{min}}\leq v_{n}\leq V_{\text{max}}$ to guide the remaining fleet to decelerate smoothly to a stop or wait at low speed.

Scenario 4: The objective function is used to minimize the time it takes for the rear vehicle to reach the stop line. Constraint $T_{(r-\text{1}{)}_{\text{1}}}+T_{s}\leq T_{r}\leq T_{g}$ ensures that the rear vehicle crosses the stop line before the green light ends while maintaining a safe following distance from the vehicle ahead. Constraint $T_{h_{\text{1}}}\geq T_{(h-\text{1}{)}_{\text{1}}}+T_{s}$ ensures that the lead vehicle maintains a proper following order relative to the vehicle ahead. Two safety distance inequalities (which calculate the difference in braking distance caused by the speed difference using acceleration a, and incorporate reaction distance and vehicle length) and the speed limit $v_{n}\leq V_{\text{max}}$ collectively ensure that no rear-end collisions or speeding occur during the acceleration process.

Scenario 5: The formula system is divided into two parts: For the front portion of the convoy that can pass through the green light after the split (the lead vehicle can pass), objective function $T_{(h-\text{1}{)}_{\text{1}}}+T_{s}\leq T_{h}\leq T_{g}$ is used to minimize the time it takes for the lead vehicle of this portion to reach the stop line. Constraint $v_{h}\leq V_{\text{max}}$ ensures that it passes before the green light ends while maintaining a safe following distance from the vehicle ahead. The safe following distance inequality uses acceleration a in conjunction with the speed limit $v_{h}\leq V_{\text{max}}$ to guide the front vehicles to accelerate through; For the remaining vehicles that cannot pass during the current green light (the rear vehicle cannot pass), the objective function is changed to $\text{max}v_{h}$, aiming to maximize the speed at which the leading vehicle of the remaining group reaches the stop line. The safety distance inequality in the constraints also uses acceleration a, incorporating reaction distance and vehicle length, while maintaining the speed limit.

Scenario 6: The objective function is used to maximize the speed of the lead vehicle at the stop line, allowing it to approach the intersection as smoothly as possible when it cannot make it through the current green light; Constraint $T_{g}\leq T_{h_{\text{0}}}+\frac{V_{\text{max}}-v_{h_{\text{0}}}}{a}+\frac{L-\frac{\overset{\text{2}}{\underset{\text{max}}{V}}}{\text{2}a}}{V_{\text{max}}}$ indicates that even with maximum acceleration, the lead vehicle cannot reach the intersection before the green light ends; constraint $T_{h}\geq T_{r}$ ensures that vehicles may only proceed after the red light ends; and constraint $T_{h}\geq T_{(h-\text{1}{)}_{\text{1}}}$ maintains the following order. The safety distance inequality uses deceleration d in conjunction with speed limit $V_{\text{min}}\leq v_{h}\leq V_{\text{max}}$ to guide the entire convoy to decelerate smoothly to the stop line and wait for the next green light cycle.

## 4. Case study

### 4.1. Data collection and processing

The paper established rigorous parameters for traffic demand and experimental scenarios to ensure the reliability and reproducibility of the experiments. Vehicle arrivals at each approach lane of the intersection follow a Poisson distribution, which effectively captures the characteristics of independent and random vehicle arrivals at urban intersections, aligning with the actual conditions of independent vehicle arrivals on urban roads. The selected traffic flow range for the experiment is 600–1,800 vehicles per hour per approach lane, corresponding to typical low-to-moderate traffic scenarios on urban roads. The intersection remains in a non-saturated state at all times, with no queue overflow or traffic gridlock, ensuring that the experimental scenario closely mirrors real-world traffic patterns. The traffic flow consists of both HDVs and CAVs. The CAV penetration rate is set between 0.1 and 0.9 to cover mixed traffic scenarios with varying levels of automation. Both vehicle types adhere to corresponding following rules: connected autonomous vehicles strictly follow speed guidance instructions, while manually driven vehicles maintain a natural driving state. This simulates a real mixed traffic environment, laying the foundation for subsequent performance validation.

In order to enhance the transparency of the experimental process and the reproducibility of results, A micro-level traffic simulation platform was developed using Python and Vissim. The relevant hardware and software configurations, along with core parameters, are shown in Table 2 and Table 3. These configurations provide a stable and reproducible simulation foundation for subsequent experimental analysis.

**Table 2:** Configuration of hardware and software facilities for experiment.

Component Category	Specification	Core Functions
CPU	Core i7-13700K	Responsible for Vissim traffic simulation scheduling, signal control logic, and serial program execution via Python.
GPU	NVIDIA RTX 4090	Accelerates data processing and batch simulation to improve algorithm performance
Memory	64 GB DDR5 6400 MHz	ensures high-speed read and write capabilities for simulation intermediate results and control commands.
Storage	2 TB NVMe SSD	Enables fast access to simulation input files, traffic demand matrices, and CSV-format trajectory/output data.
Vissim Version	PTV VISSIM 2023	Main traffic simulation platform for mixed traffic flow modeling and microscopic vehicle behavior simulation.
Python Version	3.10	implements the proposed platoon recognition and speed guidance algorithms, and interacting with Vissim

**Table 3:** Basic simulation parameter settings for the experiment.

Parameter Name	Parameter Value	Setup Instructions
Simulation Random Seed	10, 20, 30, 40, 50 (five replications per scenario)	Reduces random errors, and improve the reproducibility and stability of experimental results.
Simulation Warm-up Time	300s	Eliminates any initial state bias and ensure that traffic flow has reached a steady state before collecting data.
Total Simulation Time	3600s	Provides sufficient steady-state data to support the assessment of efficiency, energy consumption and emissions.
Time Step	0.1s	Ensures high-precision tracking of the vehicle’s trajectory, enabling smooth execution of speed control commands.
Car-Following Model	Wiedemann 74	A mixed traffic flow following model compatible with VISSIM, capable of simulating the following behaviour of CAVs and HDVs.
Standstill Distance	2.0 m	Simulates the typical following distance between vehicles when stopping at a red light at a junction.
Safety Distance Factor	1.5	Controls the following distance to ensure that the vehicle’s response to changes in speed and deceleration commands is consistent with actual traffic conditions.

**Fig 2:**
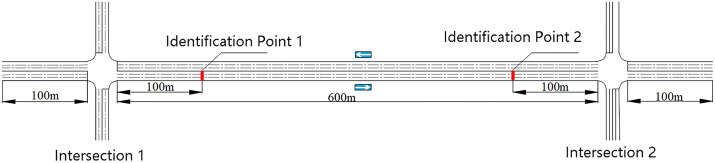
Base Map in Vissim.

First, a simulation road network was constructed using Vissim. Python was then used to retrieve basic information regarding vehicles, roads, signal controls and traffic flow through its COM interface. Subsequently, speed control instructions were sent via the COM interface to the target speed module within the Vissim network, thereby achieving vehicle speed control. Fig 2 shows the simulation platform built in Vissim. In this study, the platoon identification point was set 100 metres upstream of the intersection. The figure depicts two signalised intersections and their respective platoon identification points.

**Fig 3:**
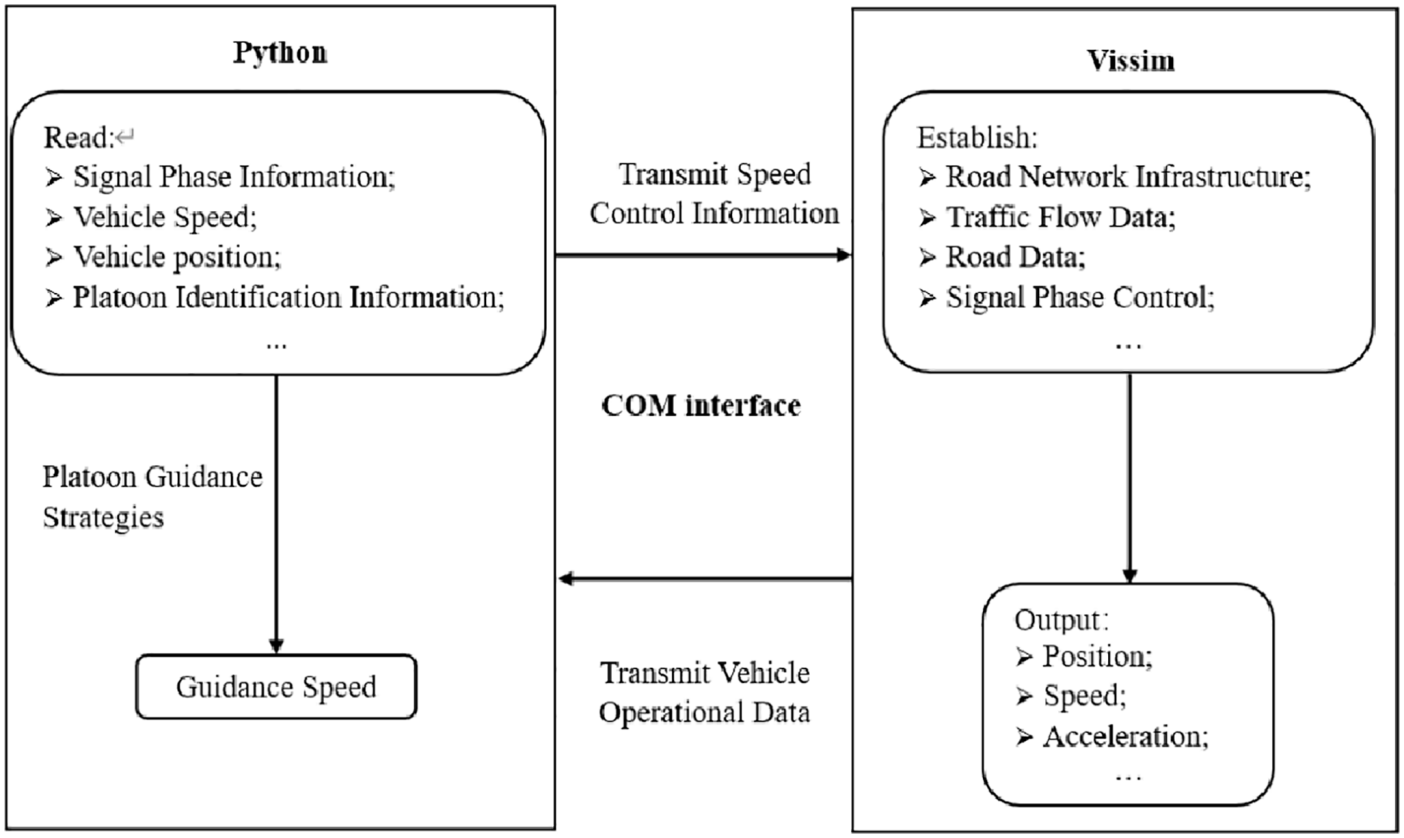
Schematic Diagram of Python and Vissim Interaction.

The speed guidance strategy outlined in this paper was implemented using Python. The algorithm has been designed to be compatible with the Vissim platform’s functional command structure, thus facilitating secondary development. The COM interface enables Python to retrieve crucial vehicle data from Vissim, including road network geometry, vehicle position, speed and acceleration. This data is then stored in the Python workspace. Starting from the vehicle identification point, the system recognises the vehicles in the platoon and combines this information with the queue conditions and the current signal phase downstream of the intersection. It then computes the recommended platoon speed and transmits it back to Vissim, achieving real-time speed guidance. The interaction between Python and Vissim is delineated in Fig 3.

The driving behavior parameters of the Vissim simulation platform were first calibrated using mixed traffic flow data collected on December 21, 2019. Through tracking and reconstruction, a total of 275,168 valid frames were obtained, comprising 5,156 frames of Tesla vehicle data and 270,012 frames of human-driven vehicle data. Based on this dataset, calibration was performed for three key driving behavior parameters in Vissim: CC0 (standstill distance), CC1 (headway time), and CC2 (traffic flow changes).

**Fig 4:**
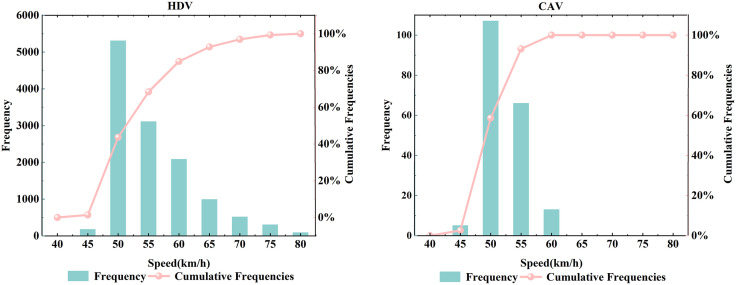
Desired Speed Distributions.

Based on the speed-density relationship, the free-flow speed of the road segment (the speed when density is zero) can be derived. Half of this speed is used as the threshold distinguishing free-flow from congested conditions. Using this threshold, the speeds of CAVs and HDVs are categorized, resulting in the desired speed distributions for CAVs and HDVs, as illustrated in Fig 4.

Calibration effectively reproduces the distinct driving behaviour characteristics of CAVs and HDVs in real-world road environments, thus laying a solid foundation for subsequent parameter optimisation and calibration using genetic algorithms in conjunction with the COM interface to reduce discrepancies between simulation and real-world data. The approach ensures that the simulation model accurately replicates real-world mixed traffic flow conditions at the data source and underlying driving rules levels. This enhances the authenticity and reliability of the simulation verification results for subsequent speed guidance strategies.

**Fig 5:**
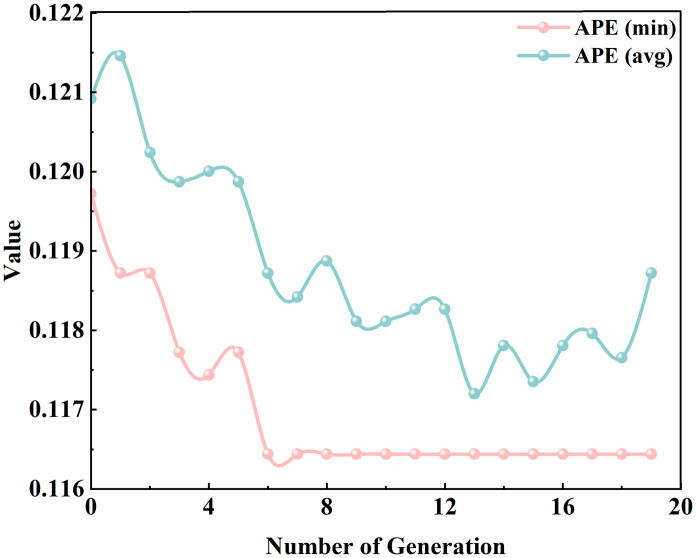
Calibration Results Based on the Genetic Algorithm.

During the calibration of driving behavior parameters using the genetic algorithm and Vissim’s COM interface, the population size was set to 15 and the number of generations to 20, resulting in a total of 300 iterations. The duration of each simulation experiment was set to 3600 seconds to ensure comprehensive testing and evaluation of the driving behavior model. This configuration allowed the algorithm to efficiently explore potential optimization paths while avoiding overfitting risks, providing sufficient time for iterative computation and adaptation to varying traffic conditions, thereby improving calibration accuracy and efficiency. To further validate the effectiveness of the calibration results, the Absolute Percentage Error (APE), calculated from the actual traffic volume on the surveyed road section and the statistically simulated traffic volume, was adopted. This metric reflects the discrepancy between model predictions and actual conditions. Fig 5 presents the calibration results, showing that the optimal solution emerged at the 7th generation, with an APE value of 12%.

**Fig 6:**
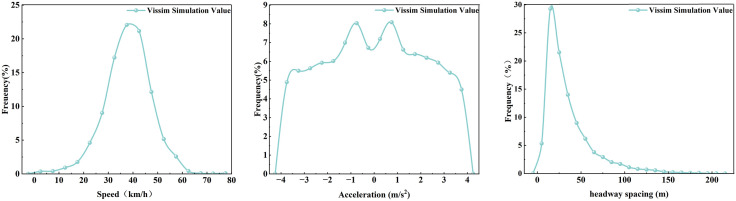
Characteristics of Driving Parameters of Vissim Built-in Car-following Model.

Moreover, in order to guarantee the precision of the experimental model, a dual strategy of field measurements and simulations was employed. A simulation platform was developed using Vissim software to account for changes in driving behaviour under a CAV environment. In order to verify whether simulations based on Vissim’s built-in car-following model could reflect the characteristic changes in driving behaviour induced by the introduction of CAV, this paper calibrated the driving parameters of Vissim’s built-in model using field data. This methodological approach permitted a precise evaluation of the model’s performance and validated its reliability in predicting and interpreting changes in complex driving environments. Subsequently, comparative analyses were conducted on the distribution characteristics of driving behaviours, including speed, acceleration, and headway, under various conditions. The frequency distribution patterns exhibited by these characteristics are indicative of the variations observed under different speeds and parameter settings, as demonstrated in Fig 6. The figure demonstrates that following the calibration of driving behaviour parameters for both CAV and HDV in Vissim using field data, the vehicle trajectory data output from the Vissim simulation effectively reflects the changes in driving behaviour within mixed traffic flows following the introduction of CAV.

**Fig 7:**
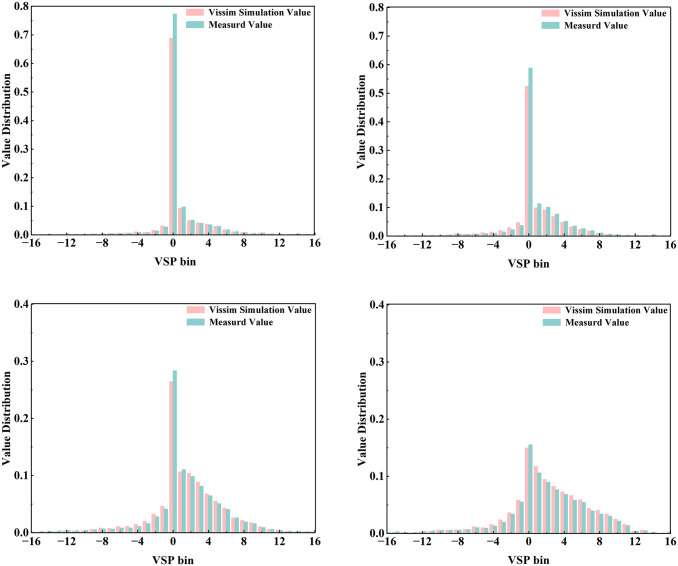
VSP Distribution Characteristics of Vissim Built-in Car-following Model.

In this paper, vehicle emissions were estimated using a calculation method based on vehicle-specific power (VSP). A comparative analysis was conducted across a range of speed intervals to compare the VSP distribution characteristics derived from VSP-based emission calculations using Vissim-simulated vehicle trajectory data with those obtained from field-measured vehicle trajectory data. This finding further validates the efficacy of the integrated field measurement and simulation approach in characterising microscopic driving behaviour parameters in CAV environments. As shown in Fig 7, after calibrating the car-following parameters for CAVs and HDVs in Vissim using cleaned and reconstructed mixed traffic flow data, the VSP distribution characteristics of the simulated vehicle trajectories based on speed intervals demonstrate a high degree of similarity to those derived from field measurements. The field VSP distribution values are calculated based on real-world emissions data accumulated over years of laboratory measurements.

It is important to acknowledge the numerous practical constraints that currently exist and hinder the widespread deployment of connected autonomous vehicles in achieving high market penetration. Firstly, the deployment costs of core hardware and software, such as on-board intelligent sensing devices and vehicle-to-infrastructure communication modules, remain high, creating significant economic barriers to large-scale adoption. Secondly, the regulatory framework for autonomous driving and unified industry technical standards are not yet fully established, and the supporting intelligent infrastructure for urban road networks remains in the stage of phased construction and iterative improvement. Thirdly, public acceptance of autonomous driving needs to be further enhanced, and the operational stability and emergency response capabilities of vehicles in complex and extreme traffic scenarios still require continuous optimization. In consideration of the contemporary development trajectory of the intelligent connected vehicle (ICV) industry, mixed traffic scenarios with low to medium CAV penetration rates are already suitable for pilot implementation and practical application. However, widespread adoption across all areas with high penetration rates will necessitate a more protracted industry incubation period. However, as technology advances, high CAV penetration rates are likely to become the norm.

Concurrently, the speed guidance strategy delineated in the paper evinces remarkable adaptability to diverse operating conditions. The system’s adaptability ensures its efficacy in diverse environments characterised by varying levels of CAV penetration, thereby addressing both the prevailing low-to-medium penetration traffic conditions and the future traffic management requirements of high-penetration scenarios, once the industry has attained full maturity. This further enhances the strategy’s engineering applicability and practical value across different stages of development.

### 4.2. Results

**Fig 8:**
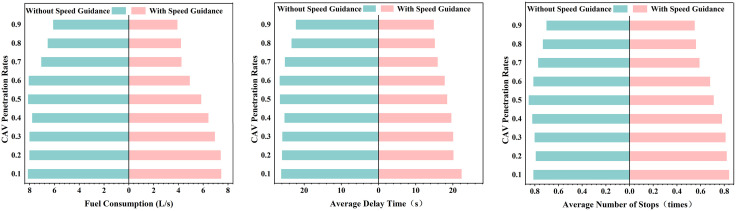
Speed Guidance Results under Different CAV Penetration Rates.

The effectiveness of the proposed platoon identification and speed guidance strategy was validated using Vissim software based on the aforementioned parameter calibration results. The platoon recognition-based speed guidance strategy was developed using Python and the Vissim software COM interface. Vehicle trajectory data output from the Vissim simulation was used to select three key performance indicators: fuel consumption per unit time, average number of stops, and average delay. A comparative analysis was conducted between scenarios with and without speed guidance under different CAV penetration rates, along with the reduction rates of the three indicators under different CAV penetration rates. The results are presented in Table 4, Fig 8 and Fig 10.

**Table 4:** Comparison of traffic performance in two scenarios.

CAV penetration rates	Without speed guidance			With speed guidance		
	fuel consumption per unit time (L/s)	average delay time (s)	average number of stops (times)	fuel consumption per unit time (L/s)	average delay time (s)	average number of stops (times)
0.1	8.130	26.11	0.81	7.446	22.32	0.84
0.2	8.004	25.89	0.79	7.404	20.11	0.82
0.3	7.998	25.81	0.80	6.932	20.03	0.81
0.4	7.782	25.23	0.82	6.414	19.55	0.78
0.5	8.112	26.45	0.85	5.832	18.43	0.71
0.6	8.058	26.53	0.81	4.906	17.77	0.68
0.7	7.050	25.11	0.77	4.234	15.89	0.59
0.8	6.528	23.33	0.73	4.196	15.11	0.56
0.9	6.092	22.17	0.70	3.913	14.83	0.55

As demonstrated in Fig 8, the fuel consumption is calculated based on the VT-Micro model [25] and represents the instantaneous fuel consumption of all vehicles in the simulation scenario. The figure indicates that, under both scenarios with and without speed guidance, the fuel consumption per unit time, average delay, and average number of stops generally decrease as the CAV penetration rate increases. In the absence of speed guidance, traffic performance demonstrates instability when the CAV penetration rate reaches approximately 0.5. This instability may be attributed to the comparable proportions of CAV and HDV, leading to noticeable conflicts as they compete for right-of-way and driving priority, thereby reducing traffic performance. Fig 8 also demonstrates that, compared to the no-guidance scenario, the scenario with speed guidance achieves lower fuel consumption per unit time, average delay, and average number of stops. Specifically, fuel consumption per unit time is reduced by 0.600 L/s–3.152 L/s, average delay is reduced by 3.79 s–9.22 s, and the average number of stops is reduced by 0.04–0.18.

**Fig 9:**
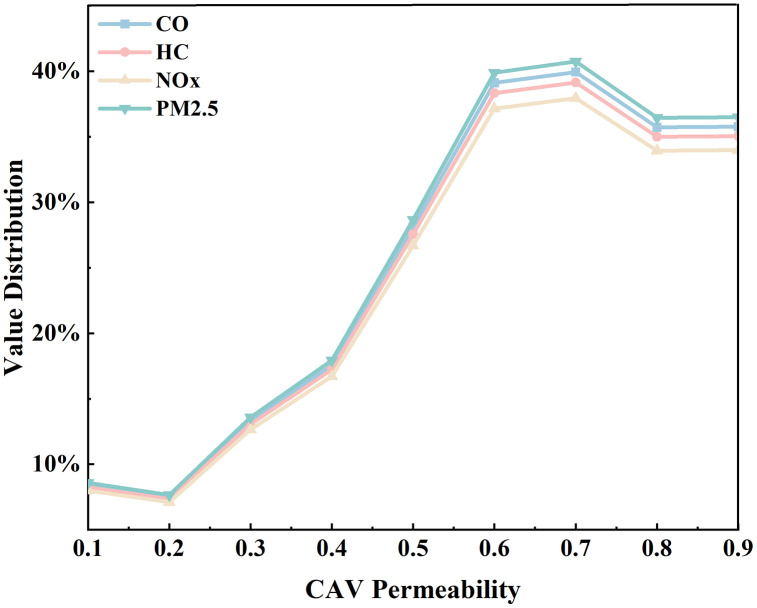
Reduction rate of Pollutant Emission Levels under Different CAV Penetration Rates.

In order to further refine the evaluation system and quantify the comprehensive optimisation effects of speed-based guidance strategies, this study also introduced an exhaust emissions evaluation dimension. The acquisition and calculation of various indicators of pollutant emission from motor vehicle exhaust emissions are based on the principles of fuel combustion, vehicle operating conditions, and standardized emission measurement systems. The present study utilises vehicle fuel consumption data to calculate and derive four typical exhaust emission indicators: carbon monoxide (CO), hydrocarbons (HC), nitrogen oxides (NOx), and fine particulate matter (PM2.5). This enhances the evaluation of strategy performance from an environmental perspective. The specific reduction rates for these indicators are demonstrated in Fig 9 It can be observed that under speed guidance strategy, typical vehicle exhaust emission indicators have all decreased, and the magnitude of these reductions generally increases as the penetration rate of CAVs rises.

**Fig 10:**
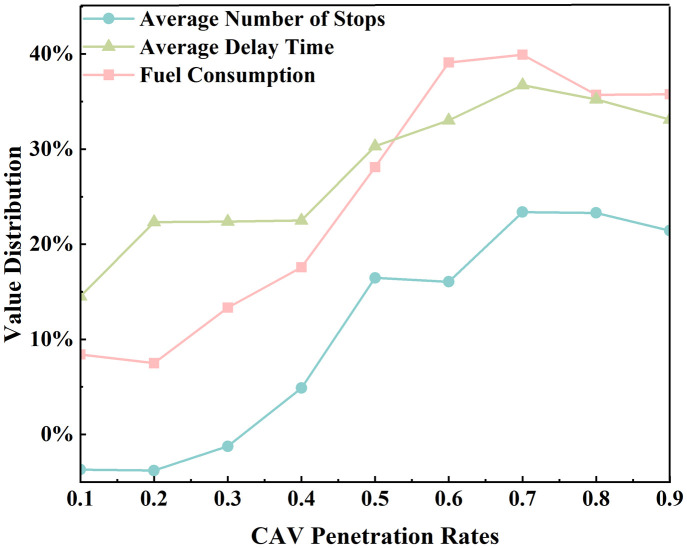
Reduction Rate of Three Indicators under Different CAV Penetration Rates.

Furthermore, the efficacy of the strategy is contingent upon the CAV penetration rate. As demonstrated in Fig 10, the reduction rates of the three metrics exhibit a gradual increase in accordance with the rising CAV penetration rate. It is notable that when the CAV penetration rate reaches 0.7, the traffic benefits under the guidance strategy are most evident, with maximum reductions in fuel consumption, delay time, and number of stops reaching 40%, 37%, and 23%, respectively.

Specifically, when the CAV penetration rate reaches 0.7, the mechanism behind the strategy’s optimal effectiveness can be analysed from the following perspectives: Firstly, with regard to the stability of traffic flow, a penetration rate of 0.7 is considered to represent a critical threshold. At this juncture, CAVs have formed a continuous convoy of sufficient scale to transmit unified speed guidance commands via vehicle-to-infrastructure (V2I) communication. This effectively suppresses the random acceleration and deceleration behaviours of manually driven vehicles, significantly reducing following disturbances in the traffic flow and minimising the coefficient of speed variation for the overall traffic flow. This provides a stable operational foundation for the convoy-based guidance strategy. Secondly, with regard to the interaction mechanism in mixed traffic, at this penetration rate, the proportion of manually driven vehicles has fallen below 30%. Consequently, the interference with the CAV convoy that is observed in such cases, including instances of forced lane changes or unexpected stops, is significantly reduced. Concurrently, the coordinated guidance signals from CAVs can still exert a certain guiding effect on surrounding manually driven vehicles, thereby achieving an effective balance between active control and passive following.

Furthermore, with regard to fleet formation characteristics, a penetration rate of 0.7 precisely meets the critical condition for forming a “stable, identifiable fleet”. This is because the number of CAVs is sufficient to form multiple continuous fleet units within the traffic flow that can be recognised by algorithms, while the proportion of manually driven vehicles is not high enough to disrupt the fleet’s continuity. At this juncture, fleet recognition accuracy attains its zenith, while the coverage of the speed guidance strategy also reaches its apogee. on the one hand, based on the law of diminishing returns, once the penetration rate exceeds 0.7: firstly, as the CAV penetration rate continues to rise, additional CAVs no longer have a significant effect on the overall stability of traffic flow or the conditions for convoy formation, and their marginal improvement effect on system performance begins to diminish; on the other hand, as the proportion of CAVs increases further, the “inefficient operational space” in the traffic flow that can be optimised (such as random starts, stops, and acceleration/deceleration disturbances caused by manually driven vehicles) continues to shrink. The physical upper limit of intersection capacity acts as a natural constraint on the downward trend in metrics such as delay, number of stops, and fuel consumption.

Moreover, in order to provide a comprehensive demonstration of the relative advantages of the Speed Guidance Strategy proposed in this paper and to enhance its persuasiveness, this study also conducts a comparative analysis with several mainstream speed guidance strategies, focusing specifically on four methods: GLOSA, MPC, Eco-Driving, and CACC. As existing speed guidance strategies (e.g., GLOSA, MPC, Eco-Driving, and CACC) exhibit significant differences in simulation parameter settings, road network structures, traffic flow conditions, vehicle composition, and application scenarios, the experimental environments and evaluation perspectives across different studies are not consistent. Therefore, this paper selects the reduction in fuel consumption – which is the most universal metric and least susceptible to experimental environment interference – as the core comparative evaluation indicator. Within the research scenario delineated in this paper and under typical operating conditions characterised by a CAV penetration rate of 0.7, the proposed fleet-based speed guidance strategy is subjected to a comparative analysis with the aforementioned four mainstream speed guidance methods. A comparative analysis is conducted by reviewing the widely accepted fuel-saving ranges of various methods in existing research [26–29] and combining them with the fuel-saving optimisation results obtained from this study’s field tests, as shown in Table 5. This clearly illustrates the improvement in energy-saving benefits and the unique advantages of this strategy, fully validating the effectiveness, rationality, and practical engineering feasibility of the proposed control method. The relevant literature references, data comparison results, and analytical discussions have all been incorporated into the thesis, effectively refining the comparative argumentation framework and further enhancing the persuasiveness of the research conclusions throughout the paper.

**Table 5:** Comparison of different speed guidance strategies.

Speed Guidance Strategy	Best Percentage Reduction in Fuel Consumption
GLOSA	27.94%
MPC	25.3%
Eco-Driving	14%
CACC	20%
The speed guidance strategy described in this paper	39.94%

As demonstrated by the comparative data, the speed-induced strategy proposed in this paper achieves an optimal fuel consumption reduction of 39.94%, which is significantly higher than the optimal fuel savings levels of mainstream methods such as GLOSA (27.94%), MPC (25.3%), Eco-Driving (14%), and CACC (20%). This fully demonstrates the significant advantages and advanced nature of this strategy in terms of energy conservation and emissions reduction.

## 5. Conclusion

This paper focuses on the problem of providing speed guidance for vehicle platoons at signalised intersections in environments involving connected and autonomous vehicles. The aim is to enhance traffic efficiency, reduce energy consumption and minimise environmental pollution through cooperative control. A systematic speed guidance strategy for mixed traffic platoons is proposed through theoretical analysis and model development. The designed strategy is validated through simulation, with its effectiveness compared under different scenarios. The key research findings are as follows:

Under both conditions with and without speed guidance, fuel consumption per unit time, average delay and average number of stops all show an overall downward trend as CAV penetration rates increase. These results demonstrate that introducing CAVs significantly enhances road capacity and efficiency, optimises traffic flow, reduces energy consumption and improves the overall sustainability and safety of the transport system.

A speed guidance strategy based on platoon recognition in connected and autonomous vehicle environments was implemented at intersections. For platoons receiving speed guidance, fuel consumption per unit time, average delay and average number of stops were significantly reduced at intersections under varying CAV penetration rates. The speed guidance strategy demonstrated the most pronounced effects when the CAV penetration rate reached 0.7, achieving the greatest reductions in fuel consumption, delay time, and number of stops: 40%, 37%, and 23% respectively.

Of course, this study has certain limitations. The research is based on idealized assumptions, which differ from real-world scenarios and do not account for objective factors such as communication delays, data packet loss, and incomplete driver compliance. Future research could incorporate factors such as communication disturbances, heterogeneity in driving behaviour and road conditions to further optimize the model and adapt it to real-world traffic environments. In addition, the research focuses solely on platoon control in single-lane, single-intersection scenarios, and the experimental road network structure is relatively simple; it does not account for complex conditions such as lane changes on multi-lane roads or traffic flow coupling. In future research, the scope could be gradually expanded to include scenarios such as multi-intersection arterial roads and complex regional road networks, with the aim of developing speed-induction strategies for platoons at complex multi-lane intersections. By calibrating the model using field-collected traffic flow and emission data, we can conduct joint simulation and field validation, and evaluate performance under varying levels of traffic demand.

This research aims to provide theoretical underpinnings and technical pathways for achieving a higher level of intelligent transportation systems by advancing theories in multi-agent collaborative optimisation and promoting the deeper integration of human, vehicle, and road infrastructure elements.

## Supporting information

S1 File 141342 pm-142853 pm_out. (XLSX)

S2 File 143839 pm-145438 pm_out. (XLSX)

S3 File 145708 pm-151257 pm_out. (XLSX)

S4 File 151355 pm-152901 pm_out. (XLSX)

S5 File 152909 pm-154419 pm_out. (XLSX)

S6 File 154854 pm-160404 pm_out. (XLSX)
